# Paeoniflorin Ameliorates Chronic Stress-Induced Depression-Like Behaviors and Neuronal Damages in Rats via Activation of the ERK-CREB Pathway

**DOI:** 10.3389/fpsyt.2018.00772

**Published:** 2019-01-14

**Authors:** Xiaoming Zhong, Guanze Li, Fengmei Qiu, Zhen Huang

**Affiliations:** ^1^Department of Chinese Medical Resources, College of Pharmacy, Zhejiang Chinese Medical University, Hangzhou, China; ^2^Pharmacy Teaching Experiment Centre, College of Pharmacy, Zhejiang Chinese Medical University, Hangzhou, China

**Keywords:** neuroprotection, paeoniflorin, chronic mild stress, ERK-CREB pathway, U0126

## Abstract

Neuronal damage is related to the onset and treatment of depressive disorders. Antidepressant-like effects have been elicited by paeoniflorin on animal models. The aim of this study is to demonstrate whether the neuroprotective effect of paeoniflorin on rats suffered from chronic unpredictable mild stress (CUMS) was regulated by the ERK-CREB signaling pathway. Results showed that paeoniflorin not only ameliorated depressive-like behavior with low locomotor activity and prolonged immobility duration in our forced swimming test but also reduced sucrose consumption. Paeoniflorin treatment decreased the degree of neuronal damage in the hippocampus of the model rats. Conversely, it markedly increased the mRNA levels of ERK1, ERK2, and CREB and the levels of ERK, p-ERK, CREB, and p-CREB protein expression in the hippocampus. Blockade of the ERK-CREB axis with the ERK-specific inhibitor U0126 repressed the neuroprotective and antidepressant-like effects of paeoniflorin on rats in the setting of chronic-mild-stress and abolished the recoveries of p-ERK mediated by paeoniflorin treatment. Thus, paeoniflorin possibly exerted a neuroprotective effect modulated by the ERK-CREB signaling pathway on CUMS-induced hippocampal damage in rats.

## Introduction

As announced by the World Health Organization, over 300 million people suffered from depressive disorders, accounting for 4.4% of the world's population in 2015. Depression is listed as the single largest contributor to global disability (1), but its mechanism and exact therapeutic drugs remain unclear.

Peony is the root of *Paeonia lactiflora* Pall., which is used to treat depression in many prescriptions of traditional Chinese medicine, including “Danggui Shaoyao San” and “Xiaoyao powder” (2–6). The total glycoside extraction of peony and its main active component paeoniflorin exert remarkable antidepressant-like effects in multiple animal models with depressive disorders (7–14). Paeoniflorin has neuroprotective effects related to depression in animal models except the CUMS model (15–18).

Pathological studies have shown neuronal damage in the hippocampus contributes to the etiology of depression and antidepressants can reverse such damage. Hippocampal atrophy is observed in patients with depressive disorders (19, 20) and changes to hippocampus are possibly caused by declined neuronal damage (21, 22). In studies on intracellular signal transduction in depression, the extracellular signal-regulated kinase–cyclic adenosine monophosphate response element binding protein (ERK-CREB) pathway plays a crucial role in the pathological change and treatment of depression, thereby regulating the growth, proliferation, and differentiation of hippocampal nerve cells, meanwhile this pathway has an important learning and memory effect on the synaptic plasticity in the brain (23, 24). However, studies have not yet to elucidate whether the ERK-CREB pathway mediates the neuroprotective effect of paeoniflorin. In the current study, the potential neuroprotection of paeoniflorin and its effect on the ERK-CREB pathway in rats with induced CUMS were assessed.

## Materials and Methods

### Drugs and Reagents

The primary antibodies against ERK (4696), p-ERK (4370), CREB (9104S), p-CREB (9198), and GAPDH (5174) were purchased from Cell Signaling Technology. The secondary antibodies, namely, goat anti-rabbit IRDye® 680CW and goat anti-mouse IRDye® 680RD, were obtained from LI-COR Biosciences, USA. TaKaRa MiniBEST Universal RNA extraction kit (Cat.9767), Prime ScriptTM RT Master Mix (Cat.RR036A), and SYBR^®^ Premix Ex *Taq* (Cat.RR420A) were procured from TaKaRa, Japan. Whole cell lysis assay (KGP250/KGP2100) and BCA protein quantitative kit were obtained from KeyGen Biotech (China) and Beijing ComWin Biotech Co., Ltd. (China), respectively. The specific primers of target genes and GAPDH (Shanghai Shenggong Co., China) were used. Paeoniflorin (purity ≥ 98%) was acquired from Huaxia Center for Certified Reference Materials (Guizhou, China, 201110). For intraperitoneal injection, paeoniflorin was dissolved in saline to the desired concentration (30 and 60 mg/kg) on the day of the test. In the present study, paeoniflorin was injected i.p. 1 h once daily before the test.

### Animals

Male Sprague Dawley rats (160–200 g) were purchased from the Animal Center of Zhejiang Chinese Medical University (SCXK2013-0016). Rats were bred at room temperature (24 ± 1°C), humidity (50 ± 10%), and day and night for 12 h, eating and drinking freely. They were bred to acclimate for 1 week before the CUMS procedures were facilitated. The laboratory animals were used according to requirements of the Ethical Committee on Laboratory Animals, Zhejiang Chinese Medical University, and experimental methods conformed to principles for protection of laboratory animals.

### Preparation of Model and Treatment

The rats were assigned to seven groups at random: control group (saline), CUMS group (saline), CUMS plus U0126 group, CUMS plus 30 mg/kg paeoniflorin group, CUMS plus 30 mg/kg paeoniflorin plus U0126 group, CUMS plus 60 mg/kg paeoniflorin, and CUMS plus 60 mg/kg paeoniflorin plus U0126 group. In the last week of CUMS, 1 μL/min U0126 was injected in the lateral ventricle once per day for 5 min. The CUMS process was carried out as described in existing literature (13) with minor modification. The rats with CUMS were exposed to varying stressors once a day for 5 weeks. These stressors included food or water deprivation for 24 h, tail nipped at 1 cm from the tip of the tail for 1 min, restraint stress for 2 h, 5 min of exposure to 45 or 4°C, and continuous illumination for 24 h. Control rats received no stimulus. Paeoniflorin and saline were injected i.p. at the same volume before each stressor was used one time per day for 5 consecutive weeks.

### Sucrose Preference Consumption Test

Sucrose preference index was detected after 5 weeks of CUMS treatment in accordance with previously described methods but with slight modifications (13). As a pretest, the rats were trained to adapt to 1% sucrose solution, which were bred individually to each rat in two bottles. After 24 h, one of the solution was converted to purified water for 24 h. Then after deprivation of water and feeding stuff for 12 h, the rats were free to obtain 1% sucrose and purified water in two bottles which were exchanged in 1 h. After 2 h, the weight of solution in every bottle was measured, and the consumed weight and rate of sucrose preference was calculated.

### Locomotor Activity Test

The locomotor activity was detected by an open-field test with slight modification (25, 26). Briefly, the test was carried out with the OFT-100 mouse and rat opening activity experiment system with a box (length of side, 100 cm), which was separated into 9 squares with weak light. The rats were individually placed at the center, and the total movement distances and the central time were recorded for 5 min with the system. The floor of the box was cleaned with water and 75% alcohol before each trial.

### Forced Swimming Test (FST)

The duration of immobility in the test was quantified by the method of Porsolt et al. (27, 28) with slightly different points. The test was performed in the FST-100 system with a transparent cylinder whose diameter was 21 cm, containing 35 cm of water with temperature at 25 ± 1°C. In brief, each rat was arranged to swim for 15 min, and then carried back to house. After 24 h, the rats were individually put in the cylinders to swim. The duration of immobility of each rat was detected for 5 min in the same system described above.

### Nissl Staining Test

The rats were sacrificed, 24 h after exposed to the last stressor of CUMS procedures. Each whole brain was rapidly dissected from the rats and flushed in ice-cold saline. The right hippocampi were separated on ice bath, immediately stored in liquid nitrogen for western blot and RT-PCR analysis. The left cerebral hemisphere was embedded in glue, and 6 μm of serial sections were performed in the coronal plane. Nissl staining were employed to observe the morphology of hippocampus and count the amount of normal nerve cells in hippocampus CA3 by microscopy. The nerve cells in hippocampal CA3 were numbered with Image-Pro Plus 6.0 software, obtaining the five-vision average to the section of the nerve cell number.

### ERK1, ERK2, and CREB mRNA Expression Levels Determined by RT-PCR

The mRNA levels were measured using SYBR RT-PCR analysis. Hippocampus samples stored in liquid nitrogen were weighed, and the extraction of total RNA was using the TaKaRa MiniBEST Universal RNA extraction kit. The total RNA was reverse-transcribed into complementary DNA with the PrimeScript^TM^ RT Master Mix and amplified in a PCR machine. The primers of the target genes and GAPDH were used, as described as follows: ERK1 F: 5′-GGGCCAAGCTTTTTCCCAAA−3′ and R: 5′- AGCCACTGGTTCATCTGTCG−3′; ERK2 F: 5′- ATCTTAAATTGGTCAGGACAAGGG−3′ and R: 5′- CTCGGAACGGCTCAAAGGAG−3′; CREB F: 5′- CTGAGGAGCTTGTACCACCG−3′ and R: 5′- CTGCTGGCATGGATACCTGG−3′; GAPDH F: 5′- ACAGCAACAGGGTGGTGAC−3′ and R: 5′- TTTGAGGGTGCAGCGAACTT−3′. Real-time quantitative PCR analysis was performed with a SYBR® Premix Ex Taq by using 7500 Real-Time PCR System with the following profile: 2 min hold at 50°C, 10 min hold at 95°C, 40 cycles of 15 s at 95°C, and 1 min at 60°C. 7500 Sequence Detection software 2.3 was used for data analysis. The relative expression levels of target genes were normalized against the level of GAPDH in the same cDNA by using the relative quantification method (2^−ΔΔ*CT*^).

### ERK, p-ERK, CREB, and p-CREB Protein Levels Detected by Western Blot

Total protein was isolated by the whole cell lysis assay and analyzed with the BCA protein quantitative kit. The proteins were separated using 10% SDS-PAGE gel and then transferred to PVDF membranes. After blocked in 5% BSA, membranes were incubated with primary antibodies (GAPDH, ERK, p-ERK, CREB, and p-CREB antibodies, 1:1000 dilution) overnight at 4°C and then exposed to the secondary antibodys (1:15000 dilution) for 1 h. Protein expression levels were quantified with Odyssey infrared scanning system software and normalized against the level of GAPDH protein as an internal control according to the previous study (29).

### Statistical Analysis

Statistical analyses were performed by SPSS 20.0. All values were presented as the mean ± SEM. Comparison between two groups was done with *t*-test or Mann–Whitney *U*-test. *P* < 0.05 was considered statistically significant.

## Results

### Effects of Paeoniflorin and U0126 on Sucrose Preference Index

**Figure 1:**
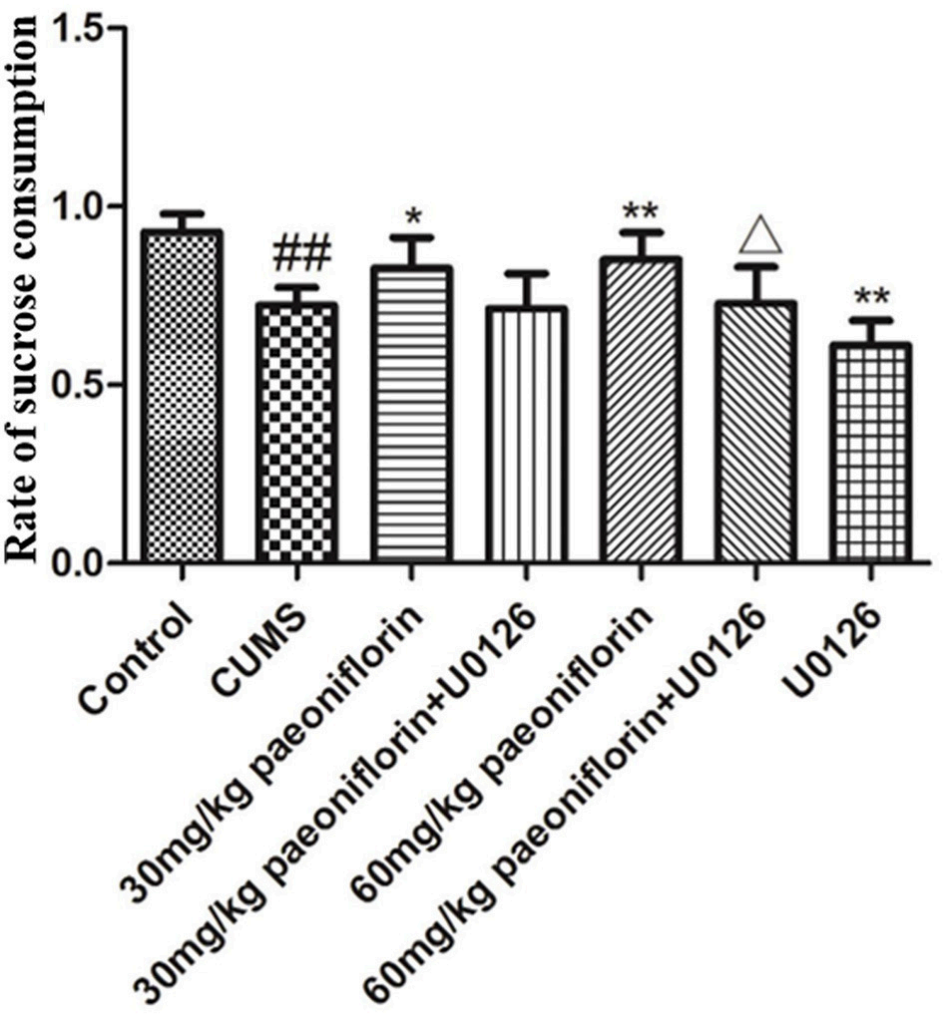
Effects of paeoniflorin on the sucrose preference index in CUMS-exposed rats (*n* = 8). ^##^*P* < 0.01 vs. control, ^*^P < 0.05, ^**^P < 0.01 vs. model group; ^Δ^*P* < 0.05 vs. 60 mg/kg paeoniflorin group.

The results are presented in Figure 1. *Post-hoc* analysis showed that the rate of sucrose preference markedly differed from each group. Exposure to CUMS significantly reduced rat sucrose consumption (*P* < 0.01 vs. control group). Paeoniflorin (30 and 60 mg/kg) markedly increased sucrose consumption in CUMS rats (*P* < 0.05, *P* < 0.01); this effect was reversed by U0126 (*P* < 0.05 when U0126 combined with 60 mg/kg paeoniflorin). Moreover, U0126 itself further decreased sucrose consumption in CUMS rats (*P* < 0.01).

### Effects of Paeoniflorin and U0126 on Locomotor Activity

**Figure 2:**
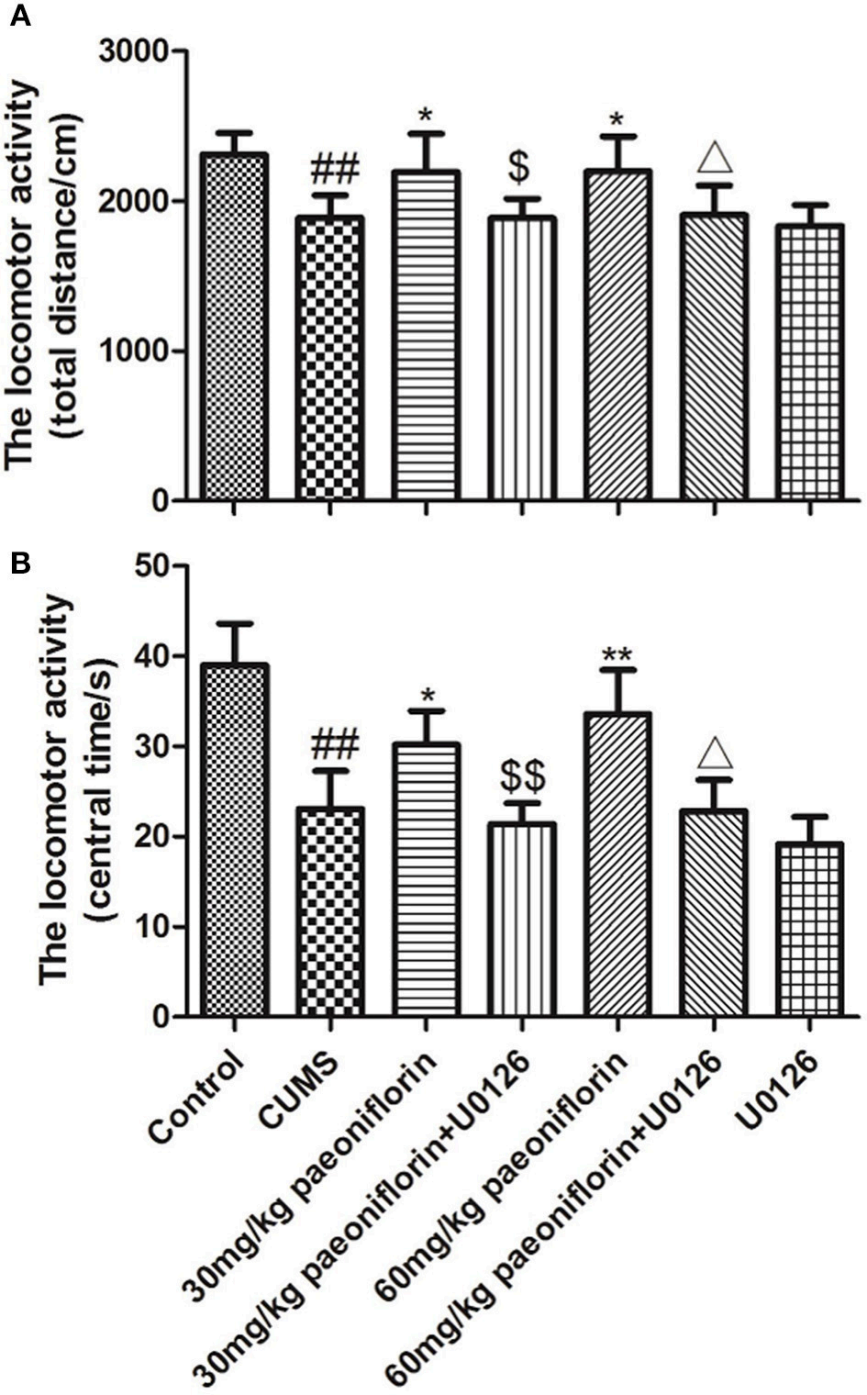
Effects of paeoniflorin on total distance **(A)** and central time **(B)** of locomotor activity in CUMS-exposed rats (*n* = 8). ^##^*P* < 0.01 vs. control; ^*^P < 0.05, ^**^P < 0.01 vs. model group; ^$^*P* < 0.05, ^$$^*P* < 0.01 vs. 30 mg/kg paeoniflorin group;^Δ^*P* < 0.05 vs. 60 mg/kg paeoniflorin group.

The total distance and the central time markedly differed among groups. *Post-hoc* analysis indicated that chronic stressors significantly reduced the total distance and the central time (*P* < 0.01), compared with the normal rats. The CUMS rats treated with paeoniflorin had an increase of the total distance and the central time vs. CUMS group (*P* < 0.05, *P* < 0.01). The U0126 group was not statistically different with the CUMS group but manifested a decrease. The paeoniflorin groups plus U0126 had a decrease of the total distance and the central time, compared with the paeoniflorin groups (*P* < 0.05, *P* < 0.01), as shown in Figure 2.

### Effect of Paeoniflorin and U0126 on Duration of Immobility in FST

**Figure 3:**
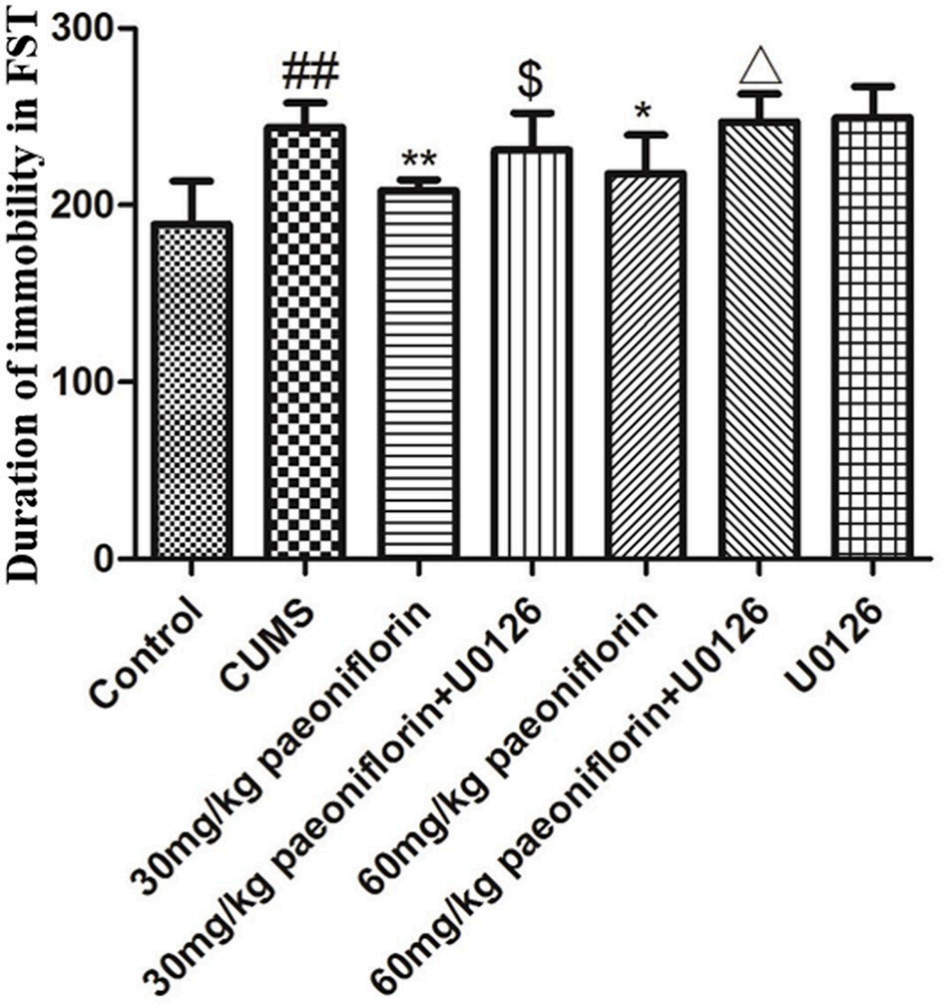
Effect of paeoniflorin on the duration of immobility of CUMS-exposed rats in FST (*n* = 8). ^##^*P* < 0.01 vs. control; ^*^P < 0.05, ^**^P < 0.01 vs. model group; ^$^*P* < 0.05 vs. 30 mg/kg paeoniflorin group; ^Δ^*P* < 0.05 vs. 60 mg/kg paeoniflorin group.

The result is depicted in Figure 3. CUMS group significantly increased the duration of immobility (*P* < 0.01) in comparison with the controls. Conversely, paeoniflorin groups reduced the total immobility time (*P* < 0.05, *P* < 0.01), and the U0126 group showed no statistical difference, though the immobility time increased. Furthermore, paeoniflorin plus U0126 groups reduced the total immobility time vs. the paeoniflorin groups (*P* < 0.05).

### Effect of Paeoniflorin and U0126 on Morphology and Number of Neuron in Hippocampus CA3

**Figure 4:**
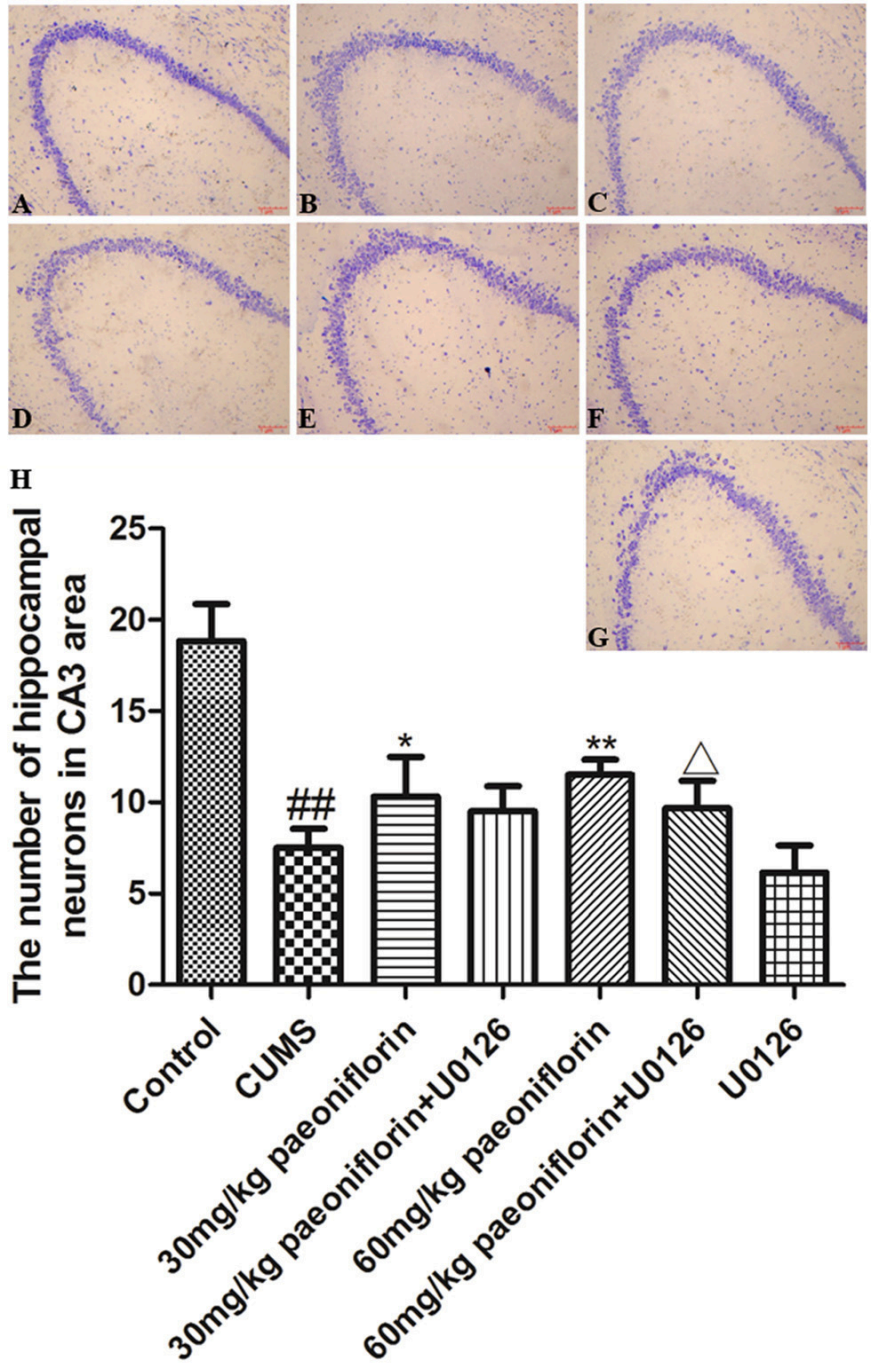
Effect of paeoniflorin on the morphology and number of hippocampal neurons in CUMS-exposed rats (*n* = 8). **(A)** control group, **(B)** CUMS group, **(C)** CUMS plus 30 mg/kg paeoniflorin group, **(D)** CUMS plus 30 mg/kg paeoniflorin plus U0126 group, **(E)** CUMS plus 60 mg/kg paeoniflorin, **(F)** CUMS plus 60 mg/kg paeoniflorin plus U0126 group, **(G)** CUMS plus U0126 group. **(H)** Effect of paeoniflorin on the number of hippocampal neurons. ^##^*P* < 0.01 vs. control; ^*^P < 0.05, ^**^P < 0.01 vs. model group; ^Δ^*P* < 0.05 vs. 60 mg/kg paeoniflorin group.

Hippocampal neurons were arranged orderly, and the Nissl substance was clear and dyed deeply in control rats (Figure 4A). In CUMS-induced rats, the hippocampal neurons were disordered and loose, and the Nissl substance was shallow-dyed and partly dissolved (Figure 4B). The U0126 group showed hippocampal nerve nucleus pyknosis, loose and disordered arrangement, austenite-shallow dye, and the degree of cell damage was similar to CUMS group (Figure 4G). The paeoniflorin groups indicated that hippocampal neurons manifested a mild disorder, partial nucleus pyknosis, austenite-shallow dye, and reduced cell damage vs. CUMS rats (Figures 4C,E). Compared with the paeoniflorin groups, the paeoniflorin plus U0126 groups aggravated cell damage (Figures 4D,F). The number of hippocampal neurons in hippocampal CA3 area was significantly reduced in the model group (*P* < 0.01) vs. control group, while paeoniflorin treatment recovered it in CUMS rats (*P* < 0.05, *P* < 0.01), and the U0126 group showed no significance. Compared with the 60 mg/kg paeoniflorin group, the number of hippocampal neurons in hippocampal CA3 area was significantly reduced in the 60 mg/kg paeoniflorin plus U0126 group (*P* < 0.05), as illustrated in Figure 4H.

### Effects of Paeoniflorin on ERK1, ERK2, and CREB mRNA Expression Levels in Hippocampus

**Figure 5:**
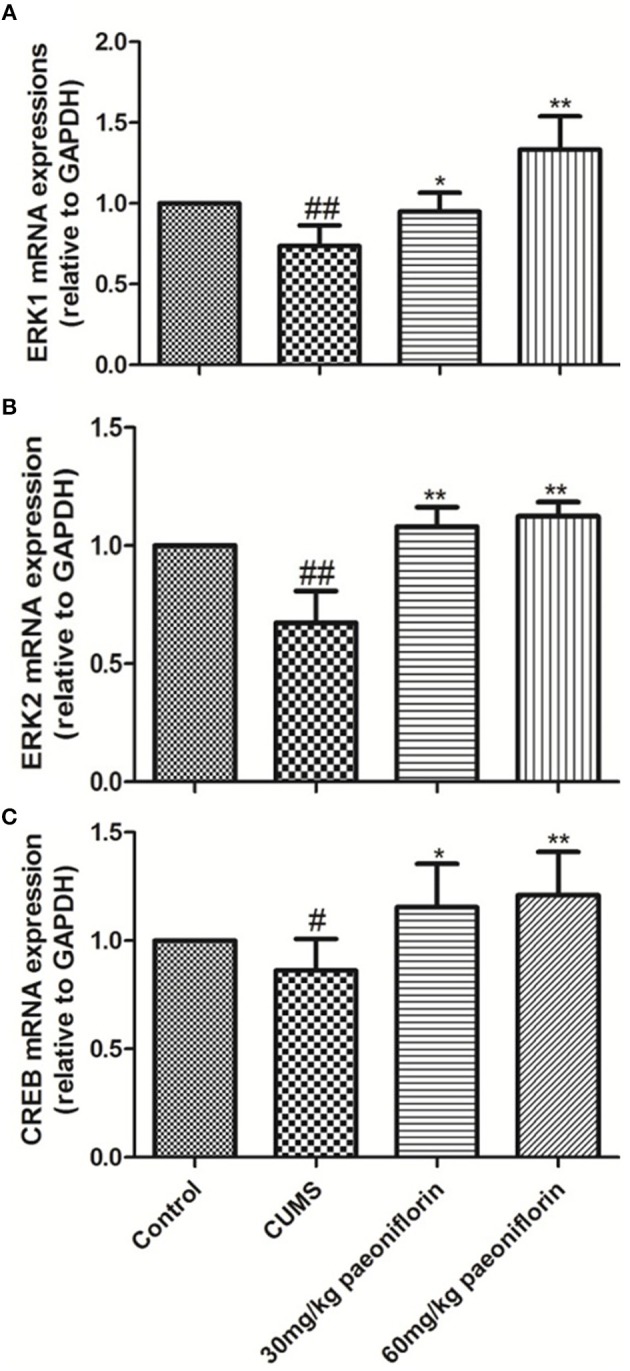
Effects of Paeoniflorin on ERK1 **(A)**, ERK2 **(B)**, and CREB **(C)** mRNA Expression Levels in Hippocampus (*n* = 8). ^#^*P* < 0.05, ^##^*P* < 0.01 vs. control; ^*^P < 0.05, ^**^P < 0.01 vs. model group.

CUMS significantly decreased the levels of ERK1, ERK2, and CREB mRNA in the hippocampus (73.6, 67.3, and 86.2%, respectively) in comparison with the controls (*P* < 0.05, *P* < 0.01). The CUMS-induced rats administrated with paeoniflorin at various doses possessed a higher ERK1, ERK2, and CREB mRNA levels in the hippocampus, compared with the CUMS-exposed rats (*P* < 0.05, *P* < 0.01), as shown in Figure 5.

### Effects of Paeoniflorin and U0126 on ERK, p-ERK, CREB, and p-CREB Protein Levels in Hippocampus

**Figure 6:**
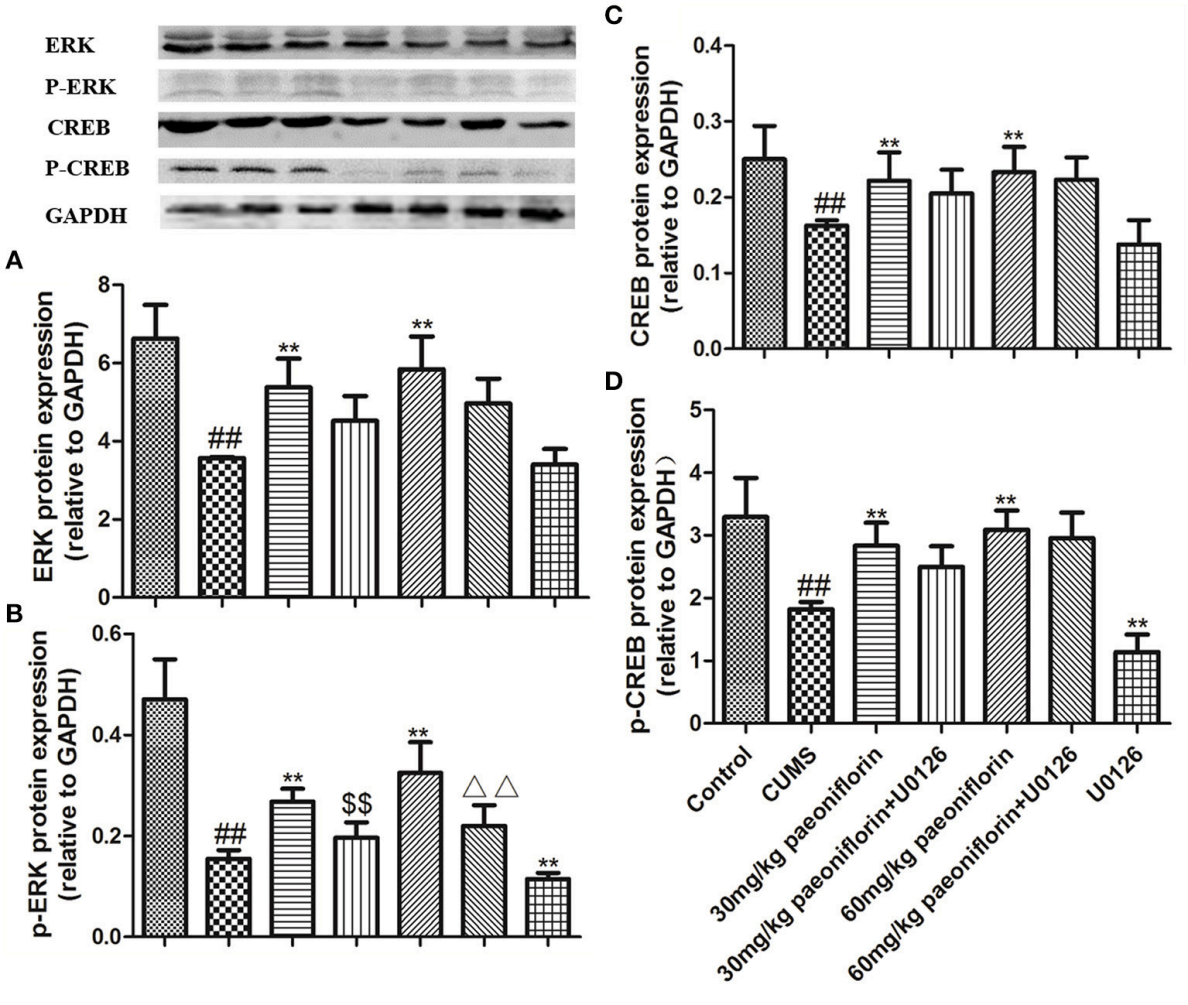
Effects of paeoniflorin on ERK **(A)**, p-ERK **(B)**, CREB **(C)**, and p-CREB **(D)** protein expression levels in the hippocampus of CUMS-exposed rats (*n* = 8). ^##^*P* < 0.01 vs. control; ^**^P < 0.01 vs. model group; ^$$^*P* < 0.01 vs. 30 mg/kg paeoniflorin group; ^ΔΔ^*P* < 0.05 vs. 60 mg/kg paeoniflorin group.

The results are shown in Figure 6. CUMS markedly decreased ERK and p-ERK protein expression levels in comparison with the control group. Paeoniflorin treatment at two doses significantly improved the expression levels of ERK and p-ERK protein compared with the CUMS-exposed rats (*P* < 0.01), while the change of p-ERK protein was prevented by the U0126. Moreover, CREB and p-CREB protein levels in the hippocampus were significantly different among groups. CUMS induced a significant decrease in the CREB and p-CREB protein (*P* < 0.01) levels, as compared with the control rats. Treatment with various dose of paeoniflorin significantly attenuated the decrease in protein levels (*P* < 0.05 and *P* < 0.01, respectively), and U0126 significantly enhanced the decrease of the p-CREB protein level (*P* < 0.01) vs. the rats with CUMS. Paeoniflorin treatment plus U0126, was not statistically significant on the levels of CREB and p-CREB protein, compared with the paeoniflorin treatment in the hippocampus.

## Discussion

Depression has become a hot spot in medical studies (30–32). The active ingredients of traditional Chinese medicine as well as the structure-activity relationship were especially researched on. The total glycoside fraction of peony exerted remarkable antidepressant-like effects in CUMS models of depression (10, 33). In the present study, paeoniflorin could clearly increase the sugar preference index, total movement distance, and the central movement time and reduce the immobility time in FST of CUMS model rats, which indicated that paeoniflorin could ameliorate the depression-like behaviors. Additionally, paeoniflorin could reduce the structural damage in the hippocampus and improve the pathological injury of brain tissues in the rats with CUMS by the Nissl staining experiment, prompting the antidepressant effects of paeoniflorin.

Based on the previous activity screening experiments, the present study clarified regulation mechanism of signaling pathway of paeoniflorin in rats with CUMS. Our previous researches (18) revealed that total glycoside fraction of peony could clearly increase BDNF protein and gene expression levels in the hippocampus of the CUMS rats and the cortisone-induced depression model rats. In fact, the neuroprotective effects of BDNF were based on the process that BDNF combined with TrkB to initiate multiple signaling pathways. The ERK-CREB signaling pathway possesses an important effect in mediating cell reaction procedures and participates in various physiological effects, such as signal transmission and identification between cells, and cell growth and development. The phosphorylation of ERK can activate its downstream signaling molecule CREB expression, which has been proposed to partly response to the expression of most cAMP-regulated genes, including BDNF. The subsequent release of target proteins can promote neuronal survival, thereby exerting antidepressant effects (34). Our experiment selected ERK-CREB signaling pathway as a breakthrough point, used specific inhibitor U0126 to block the pathway, and observed the spontaneous activities and the change of the key molecular effects of the signaling pathway in rats with CUMS. Based on the RT-PCR and Western blot experiments, paeoniflorin could markedly increase the ERK1, ERK2, and CREB mRNA expression levels and the ERK, p-ERK, CREB, and p-CREB protein expression levels in the hippocampus of CUMS-exposed rats, while the activation of p-ERK protein by paeoniflorin in model rats could be blocked by U0126. Futhermore, U0126 repressed the neuroprotective and antidepressant-like effects of paeoniflorin on rats in the setting of CUMS. This research indicated that the neuroprotection of paeoniflorin may play a role of antidepressant by activating the ERK-CREB pathway. This signaling pathway is also activated by multiple anti-depressant drugs, such as Xingnao Jieyu Decoction, memantine, and so on (34, 35), which suggests that the ERK-CREB pathway plays a key role in the treatment of depression.

In summary, paeoniflorin could alleviate the depression of rats with CUMS and ameliorate the pathological injury of nerve cells in the hippocampus, indicating that the antidepressant effect and protective effect of paeoniflorin on hippocampal nerve cells might be mediated by ERK-CREB signaling pathway, which are the downstream pathway of BDNF. However, understanding a signaling pathway is inadequate to study the pathogenesis of depression. We should seek other effective multiple signaling pathways involved in the pathogenesis of depression and explore cross connections and interactions among various pathways.

## Nomenclature

### Resource Identification Initiative

The antibodies against ERK: Cell Signaling Technology Cat# 4696, RRID:AB_390780.

The antibodies against p-ERK: Cell Signaling Technology Cat# 4370, RRID:AB_2315112.

The antibodies against CREB: Cell Signaling Technology Cat# 9104S, RRID:AB_10691832.

The antibodies against p-CREB: Cell Signaling Technology Cat# 9198, RRID:AB_2561044.

The antibodies against GAPDH: Cell Signaling Technology Cat# 5174, RRID:AB_10622025.

## Author Contributions

XZ contributed to data collection and drafted the article. GL contributed to preparation of animal models and medicine administration. FQ contributed to coordination of experiments, data collection, and statistical analysis. ZH designed the study and revised the manuscript for important intellectual content. All authors contributed to and approved the final manuscript, and agreed to be accountable for all aspects of the work.

### Conflict of Interest Statement

The authors declare that the research was conducted in the absence of any commercial or financial relationships that could be construed as a potential conflict of interest.
